# Novel *APC *mutations in Czech and Slovak FAP families: clinical and genetic aspects

**DOI:** 10.1186/1471-2350-8-16

**Published:** 2007-04-05

**Authors:** Jitka Stekrova, Martina Sulova, Vera Kebrdlova, Katerina Zidkova, Jaroslav Kotlas, Denisa Ilencikova, Kamila Vesela, Milada Kohoutova

**Affiliations:** 1 Institute of Biology and Medical Genetics of the 1st Faculty of Medicine and General Teaching Hospital, Charles University, Albertov 4, Prague 2, 128 00, Czech Republic; 2 National Cancer Institute, Department of Cancer Genetics, Bratislava, Slovak Republic

## Abstract

**Background:**

Germline mutations in the adenomatous polyposis gene (*APC*) result in familial adenomatous polyposis (FAP). FAP is an autosomal dominantly inherited disorder predisposing to colorectal cancer. Typical FAP is characterized by hundreds to thousands of colorectal adenomatous polyps and by several extracolonic manifestations. An attenuated form of polyposis (AFAP) is characterized by less than 100 adenomas and later onset of the disease.

**Methods:**

Here, we analyzed the *APC *gene for germline mutations in 59 Czech and 15 Slovak FAP patients. In addition, 50 apparently *APC *mutation negative Czech probands and 3 probands of Slovak origin were screened for large deletions encompassing the *APC *gene. Mutation screening was performed using denaturing gradient gel electrophoresis and/or protein truncation test. DNA fragments showing an aberrant electrophoretic banding pattern were sequenced. Screening for large deletions was performed by multiplex ligation dependent probe amplification. The extent of deletions was analyzed using following microsatellite markers: D5S299, D5S82, D5S134 and D5S346.

**Results:**

In the set of Czech and Slovak patients, we identified 46 germline mutations among 74 unrelated probands. Total mutation capture is 62,2% including large deletions. Thirty seven mutations were detected in 49 patients presenting a classical FAP phenotype (75,5%) and 9 mutations in 25 patients with attenuated FAP (36%). We report 20 novel germline *APC *mutations and 3 large deletions (6%) encompassing the whole-gene deletions and/or exon 14 deletion. In the patients with novel mutations, correlations of the mutation localization are discussed in context of the classical and/or attenuated phenotype of the disease.

**Conclusion:**

The results of the molecular genetic testing are used both in the establishment of the predictive diagnosis and in the clinical management of patients. In some cases this study has also shown the difficulty to classify clinically between the classical and the attenuated form of FAP according to the established criteria. Interfamilial and/or intrafamilial phenotype variability was also confirmed in some cases which did not fit well with predicted genotype-phenotype correlation. All these findings have to be taken into consideration both in the genetic counselling and in the patient care.

## Background

Germline mutations in the adenomatous polyposis coli (*APC*) gene have been shown to result in the familial adenomatous polyposis (FAP) syndrome. FAP (OMIM# 175100) is an autosomal dominantly inherited disorder predisposing to colorectal cancer (CRC) and accounts for 1% of all CRC cases. Classical FAP is characterized by hundreds to thousands of colorectal adenomatous polyps developing during the second decade of life. Some of these polyps progress to colorectal carcinoma if the whole colon is not removed as a prophylactic intervention. Several extracolonic manifestations are frequently presented in FAP patients, including gastric and duodenal adenomas, desmoid tumors, congenital hypertrophy of the retinal pigment epithelium (CHRPE), osteomas, dental abnormalities, epidermoid skin cysts and malignancies of other organs. In addition to the classical form of FAP, there are patients with an attenuated form of polyposis (AFAP) characterized by less than 100 adenomas and later onset of the disease. The *APC *gene located at chromosome 5q21 was identified in 1991 [1,2] and more than 600 germline mutations have been reported to date. The majority of the *APC *germline mutations are frameshift, nonsense or splice site mutations in the 5'half of the gene resulting in a truncated protein. The APC protein is a part of the wnt signalling pathway and contains several important domains involving in processes such as cell cycle regulation, cell adhesion and apoptosis [3].

A number of screening methods have identified germline *APC *mutations in 60–80% of all FAP patients [4]. Several causations of the disease have been supposed in *APC *mutation negative patients: (i) Recent studies have revealed germline mutations in the base excision repair *MUTYH *gene, leading to the *MUTYH *associated polyposis (MAP) [[5,6] and [7]]. MAP is an autosomal recessive form of polyposis with manifestation of AFAP in the majority of the cases. (ii) In several studies, large deletions have been found in the 5q21 region including the *APC *gene [[8,9] and [10]]. These large deletions cannot be detected by conventional mutation screening methods. (iii) Mutations in both introns and regulatory regions of the *APC *gene can escape the detection. (iv) Mutations in not yet identified genes can be considered as the reason of the disease.

The objectives of this report were: (i) to complete and to characterize germline *APC *mutations in the Czech and partly in the Slovak population; (ii) to asses whether the large *APC *deletions might explain the FAP occurrence in a part of Czech *APC *mutation negative patients; and (iii) to determine genotype – phenotype associations.

## Methods

### Patients

Fifty nine unrelated Czech patients and 15 unrelated Slovak patients with FAP were screened for *APC *germline mutations. The set of patients included 36 Czech and 13 Slovak cases presenting with a classical FAP and 23 Czech and 2 Slovak cases with an AFAP phenotype. All of them came from various health institutions in Czech and Slovak Republic and were referred on the basis of colonoscopic findings and/or positive family history. The study has been performed with the approval of Ethic Committee of the General University Hospital in Prague. Written informed consent was obtained from each probands before genetic testing. Together with our previous studies [11,12] the set of 136 unrelated patients with FAP were analyzed. Fifty apparently *APC *mutation negative Czech probands from this set of patients (24 probands from this paper and 26 patients from our previous studies) and 3 probands of Slovak origin were screened for large deletions encompassing the *APC *gene.

Phenotype classification was based on two criteria: the number of polyps and the age at diagnosis of FAP. The phenotype was classified as typical FAP when the patient presented with > 100 polyps in the second and/or third decade of life with the ocurrence of CRC in or before the fourth decade of life. The attenuated phenotype was characterized by the occurrence of < 100 of polyps in the third or fourth decade of life and later occurrence of the CRC.

### Mutation screening

Genomic DNA was isolated from peripheral blood lymphocytes by the salting-out procedure [13]. The coding region and intron – exon boundaries of the *APC *gene were screened for mutations using DGGE (Denaturing Gradient Gel Electrophoresis) and/or PTT (Protein Truncation Test). All patients were examined by very sensitive DGGE method while only a small part of patients was examined by PTT simultaneously. For DGGE analysis exons 1–15 were amplified using the set of GC-clamped primers and PCR conditions as previously reported by Olschwang et al. [14,15]. The protein truncation test (PTT) was performed using TnT T7 Quick for PCR DNA system (Promega). The test was used for screening of exon 15 which was amplified from genomic DNA in four overlapping segments as described in detail by van der Luijt and Meera Khan [16]. The coupled in vitro transcription-translation was performed in the presence of S^35 ^– methionin. After translation, the products were separated on 15% SDS-polyacrylamide gel and detected by fluorography. All DGGE and PTT fragments showing an aberrant electrophoretic banding pattern were sequenced in both directions using an automatic ABI Prism™ 310 Genetic Analyzer (PE Applied Biosystem) in accordance with the manufacturer's instructions. All mutations found were confirmed by repeated analysis of the second independent blood taking.

Screening for large deletions was performed by MLPA (Multiplex Ligation Dependent Probe Amplification) method using the MLPA kit SALSA P043 (MRC Holland). Briefly, the protocol consists of DNA denaturation, hybridisation of the SALSA probes to each target sequence, followed by a ligation reaction. The target sequences are amplified in a multiplex PCR and the PCR products are separated on ABI Prism 310 Genetic Analyzer accordance to the manufacturer's instruction.

To identify the extent of gross deletions we applied following microsatelite markers: D5S299, D5S82, D5S134 and D5S346. The sequence from centromere to telomere is D5S299, D5S82, D5S134, the *APC *gene, D5S346, the *MCC *gene. DNA was amplified by using following primers:

D5S299: S299F: GCTATTCTCTCAGGATCTTG

S299R: GTAAGCCAGGACAAGATGACA

D5S82: S82F: ATCAGAGTATCAGAATTTCT

S82R: CCCAATTGTATAGATTTAGAA

D5S134: S134F: ACATCTCCAATATACCCCCCTCTCTCTTTC

S134R: TCCTCTGTGGTTGGTGAAATTGCACC

D5S346: S346F: ACTCACTCTAGTGATAAATCGGG

S346R: AGCAGATAAGACAGTATTACTAGTT

The PCR products were separated on 9% polyacrylamid gel for 5 hours and the gels were stained with ethidium bromide and photographed under UV light.

## Results and discussion

Mutation analysis of the *APC *coding region resulted in the identification of 46 pathogenic mutations including large deletions in 74 unrelated Czech and Slovak patients (62,2%). Thirty seven germline mutations were detected in 49 patients with classical FAP (75,5%) and 9 mutations in 25 patients with AFAP (36%). Twenty mutations were novel. The summary of the mutations identified in this set of Czech and Slovak patients is given in Tab. 1.

**Table 1:** *APC *germline mutations in Czech and Slovak FAP patients

FAMILY	PHENOTYPE	NUCLEOTIDE CHANGE	CONSEQUENCE	REFERENCE
**147**	**AFAP**	**c.220G>T**	p.Glu74X (*)	**Current paper**
**229**	**AFAP**	**c.230T>G**	**p.Leu77X**	**Current paper**
**177**	**AFAP**	**c.288_289insCC**	**p.Gly97ProfsX29**	**Current paper**
**176**	**AFAP**	**c.505_509delATAGA**	**p.Ile169X**	**Current paper**
150	FAP	c.517_520delCCTT	p.Pro173X	[20]
**179**	**AFAP (V)**	**c.531+1G>T**	*	**Current paper**
155	FAP	c.637C>T	p.Arg213X	[25]
208	FAP	c.637C>T	p.Arg213X	[25]
**164**	**FAP**	**c.645+1G>T**	*	**Current paper**
144	FAP	c.646C>T	p.Arg216X	[26]
162	FAP	c.646C>T	p.Arg216X	[26]
174	FAP	c.646C>T	p.Arg216X	[26]
S8	FAP	c.646C>T	p.Arg216X	[26]
**S7**	**FAP (L)**	**c.759_787del29**	**p.His255ArgfsX11**	**Current paper**
S14	FAP (L)	c.834+1G>A	*	[14]
**226**	**FAP**	**c.880delT**	**p.Ser294LeufsX11**	**Current paper**
211	AFAP	c.994C>T	p.Arg332X	[27]
**262**	**AFAP**	**c.1102_1103delGT**	**p.Val368IlefsX9**	**Current paper**
**220**	**AFAP**	**c.1111G>T**	**p.Gly371X**	**Current paper**
**202**	**FAP**	**c.1251delT**	**p.Cys417TrpfsX37**	**Current paper**
**215**	**FAP (L)**	**c.1284delA**	**p.Glu428AspfsX26**	**Current paper**
**S16**	**FAP**	**c.1370C>G**	**p.Ser457X**	**Current paper**
173	FAP	c.1411G>T	p.Gly471X	[12]
**S1**	**FAP**	**c.1624C>T**	**p.Gln542X**	**Current paper**
**157**	**FAP**	**c.2024delC**	**p.Thr675LysfsX2**	**Current paper**
**145**	**AFAP (V)**	**c.2031_2034delCAGT**	**p.Ser678MetfsX39**	**Current paper**
**203**	**FAP**	**c.2031_2034delCAGT**	**p.Ser678MetfsX39**	**Current paper**
**233**	**FAP**	**c.2390delG**	**p.Gly797ValfsX23**	**Current paper**
171	FAP	c.2626C>T	p.Arg876X	[28]
S15	FAP (L)	c.2800_2803delACTT	p.Tyr935IlefsX19	[29]
195	FAP	c.2805C>A	p.Tyr935X	[30]
242	FAP	c.2805C>A	p.Tyr935X	[30]
**206**	**FAP**	**c.2977_2980dupAAGT**	**p.Phe994X**	**Current paper**
**S10**	**FAP**	**c.3413delA**	**p.Asp1138ValfsX27**	**Current paper**
153	FAP	c.3471_3474delGAGA	p.Glu1157AspfsX7	[31]
181	FAP	c.3471_3474delGAGA	p.Glu1157AspfsX7	[31]
**240**	**FAP**	**c.3513dupT**	**p.His1172SerfsX7**	**Current paper**
225	FAP	c.3786T>A	p.Tyr1262X	[4]
183	FAP	c.3927_3931delAAAGA	p.Glu1309AspfsX4	[25]
205	FAP	c.3927_3931delAAAGA	p.Glu1309AspfsX4	[25]
218	FAP	c.3927_3931delAAAGA	p.Glu1309AspfsX4	[25]
248	FAP	c.3927_3931delAAAGA	p.Glu1309AspfsX4	[25]
S12	FAP	c.4666dupA	p.Thr1556AsnfsX3	[32]

cDNA numbering is based on reference sequence: GenBank NM_000038, +1 corresponds to the A of the ATG translation initiation codon.

Novel mutations in **boldface **type. S – Patient of Slovak origin, V – intrafamilial variable phenotype, L – late onset of the disease; > 100 polyps. * Mutations can affect splicing

Our mutation detection rate is consistent with previous reports. Using standard mutation analysis, 20–30% of classical FAP patients have no detectable *APC *mutation. The detection rate can be increased using other tests such as MLPA analysis, MAMA (Monoallelic Mutation Analysis) and so on. In our study, it is quite likely that mutations in regulation and/or in noncoding regions are missed. It was shown that newer diagnostic method such as MAMA [17] combined with standard genetic testing can identify *APC *mutations in > 95% FAP patients. Using this method, two *APC *alleles can be examined independently. Reduced expression in mutant allele can be demonstrated at protein level. In a set of *APC *mutation negative patient, biallelic mutations in *MUTYH *gene can also be responsible for polyposis same as mutations in not yet identified genes.

In 59 unrelated Czech patients, standard mutation analysis of the *APC *coding region revealed 35 germline mutations. Among the 36 probands presenting a classical FAP phenotype, *APC *point mutations were detected in 26 patients (72,2%). Nine mutations (9/23 probands) were identified in patients with AFAP (39,1%). A total of 26 different mutations were detected, including 16 novel ones (detected in 17 patients), not previously reported. Of the 16 newly described mutations, fourteen were truncating and two ones affected donor and/or acceptor splice site. Using MLPA analysis, deletion screening revealed large deletions in 3 of the 50 Czech patients (6%) with no *APC *point mutation. Together with our previous studies [11,12] ninety two germline mutations including large deletions were detected in 136 unrelated Czech patients, thus the mutation detection rate was 67,6 %. In this combined sample, the mutation detection rate in classical FAP is 77,2% (78/101) and 40% (14/35) in attenuated FAP. In accordance with previous studies, the most common mutation was the codon 1309 5 bp deletion (10%) [4]. Substitution c.2805C>A at codon 935 (7%) was also quite frequent. However the frequency of the deletion at codon 1061 seems to be lover (3%) than referred data [3].

In a set of Slovak probands, eight germline mutations were discovered in 13 patients with classical FAP (61,5%), including four novel mutations, not previously reported (Tab. 1). No mutation was detected in 2 patients with AFAP. Large deletions were screened in 3 Slovak FAP patients with no *APC *point mutation. MLPA analysis revealed no large deletion.

### Novel mutations

**Figure 1:**
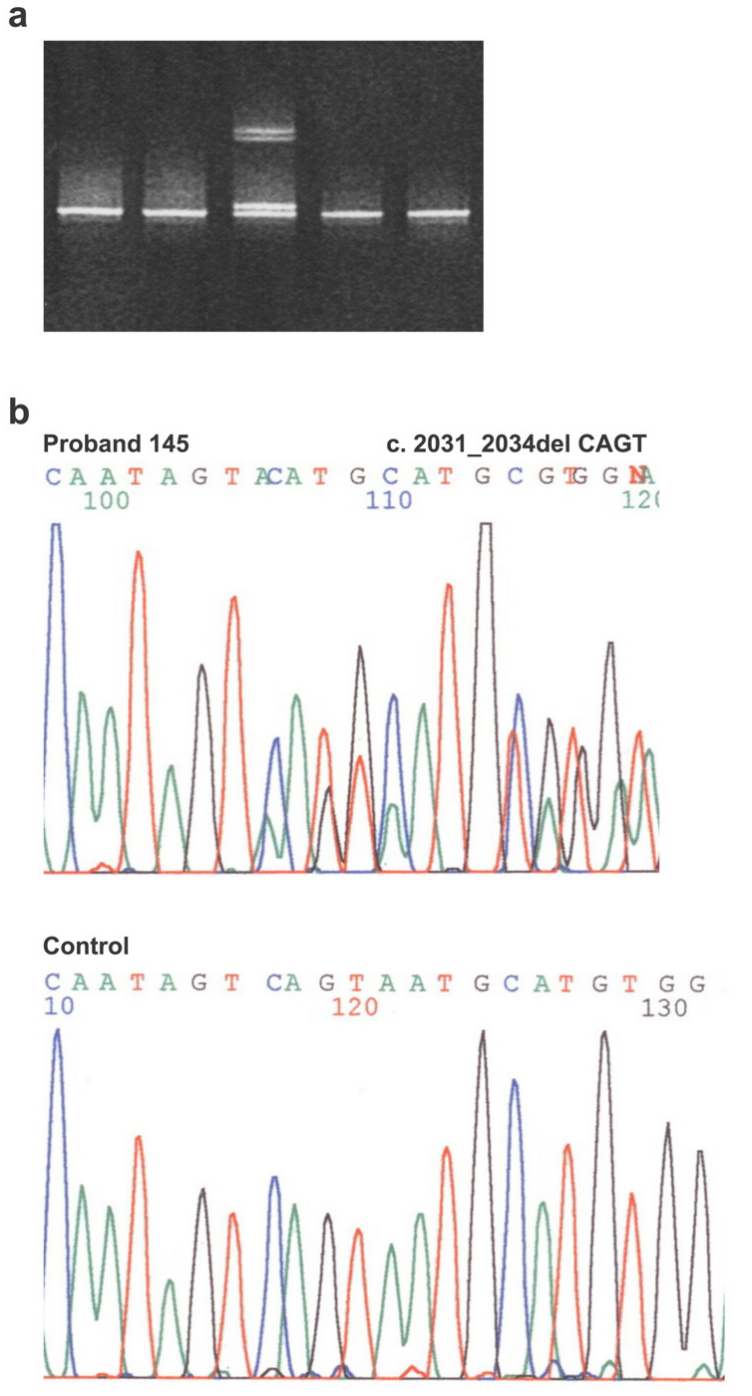
Analysis of *APC *germline mutations of exon 15A. **a**. Denaturing Gradient Gel Electrophoresis PCR products of exon15A of FAP patients. Lane 3 represents heterozygous proband of the family 145. **b**. Sequencing pattern of part of the exon 15A (forward) in proband of the family 145 with heterozygous deletion CAGT at nucleotide position 2031_2034 (p.Ser678MetfsX39) and control.

Out of the **17 **Czech patients with identified novel mutations, 9 patients presented a classical form of FAP and 8 patients suffered from an attenuated form of the disease. In concordance with published reports, the AFAP phenotype was associated with mutations in exons 2, 3, 4 and 9 (Tab. 1). Different phenotypes were found in two unrelated patients (probands 145 and 203) with the same 4 bp deletion in exon 15 (Fig. 1). The proband 203 presented classical FAP with early onset of polyposis and colorectal cancer while the proband 145 was diagnosed for polyposis in the fifth decade of life and he underwent the colectomy at the age 58. His mother was diagnosed for CRC at the age of 61 and the diagnosis of CRC occurred also in his 3 brothers and his sister (at the age of 39, 47, 68 and 31 respectively). The intrafamilial variations in the FAP phenotype are known and it is supposed that modifying genes and/or environmental factors could play a role in determining the phenotype.

The mutation found in the proband 147 (c.220G>T) leads to a creation of stop codon (p.Glu74X) keeping the dimerization domain in the APC protein. However, this single base substitution affected the last nucleotide of the exon 2 and consequently the aberrant splicing cannot be excluded (77% likelihood). The proband is classified to have AFAP because first symptoms displayed as CRC at 62 of age. This type of mutation was also demonstrated in his sister (60 years old), both brothers (54 and 62 years old) and two daughters (35 and 38 years old). Now, colonoscopic examinations are performed in his relatives.

Patients 229 and 177 with mutation in exon 2 and 3 respectively presented with an attenuated phenotype (with about 100 adenomas diagnosed at age of 62 and 63 years respectively and with no evidence of carcinoma).

In the case of the proband 179, a single base pair change at the conserved splice donor +1 position of intron 4 (c.531+1G>T) was identified predicting the splice defect. The proband is classified as an attenuated form of FAP on the basis of the polyp number: only three polyps developed when the proband was 22 years old. However, his brother can display a phenotype with more likely intermediate features between the attenuated and classic forms of FAP with respect to the onset of the disease and number of polyps: he was diagnosed for polyp occurrence when he was 15 years old and he developed < 100 polyps at this age. The proband's father was diagnosed for colorectal cancer at the age 41 and he agreed for colectomy. Two additional reports describe mutations in intron 4 of the *APC *gene that result in the splice defect. An insertion of a single thymidine (c.531 +2_531 +3insT) [18] and two substitutions at the donor splice site and in its neighbourhood (c.531+1G>A, c.531+5G>C) [19] were identified in American and in two Finish families. The phenotypes of these patients are consistent with the attenuated FAP phenotype. Our and previous results confirmed the suggestion that this *APC *region is prone to intronic mutations leading to splice defect [18].

A phenotype of our proband 179 and his brother resembles the recently described AFAP syndrome with atypical disease course caused by a CCTT deletion in exon 4 (codon 173) [20]. In this case, the proband revealed a low number of polyps (approximately 30) at the age of 17 and his father and grandfather died due to colorectal cancer at 35 and 40 years of age. Our proband (176) with the deletion in exon 4 presented attenuated form of FAP with the onset of the disease during the fifth decade of life (diagnosed for CRC at age 47). His son developed about 100 polyps at age 21 years. However, the phenotype of the proband (150) showing the same mutation described by Plawski [20] corresponds rather to classic course of FAP with occurrence CRC at age of 37. Unfortunately, the number of polyps is unknown. An additional report describes a nonsense mutation in codon 161 (exon 4) [21] leading also to a phenotype with features between classic and attenuated forms of FAP (later onset and more than 100 colorectal polyps). Classical form of FAP is described in two patients with mutations at the invariant splice acceptor site of exon 4 [24] . Both patients presented with > 100 adenomas at age of 32 – 35 years. Mutations at 5'end of the *APC *gene (especially in exons 3 and 4) were originally described to be associated with the attenuated phenotype. However, it appears that the mutations located in exon and intron 4 lead to the greater phenotype variability resulting in a difficult classification between classical and attenuated forms of the disease.

**Figure 2:**
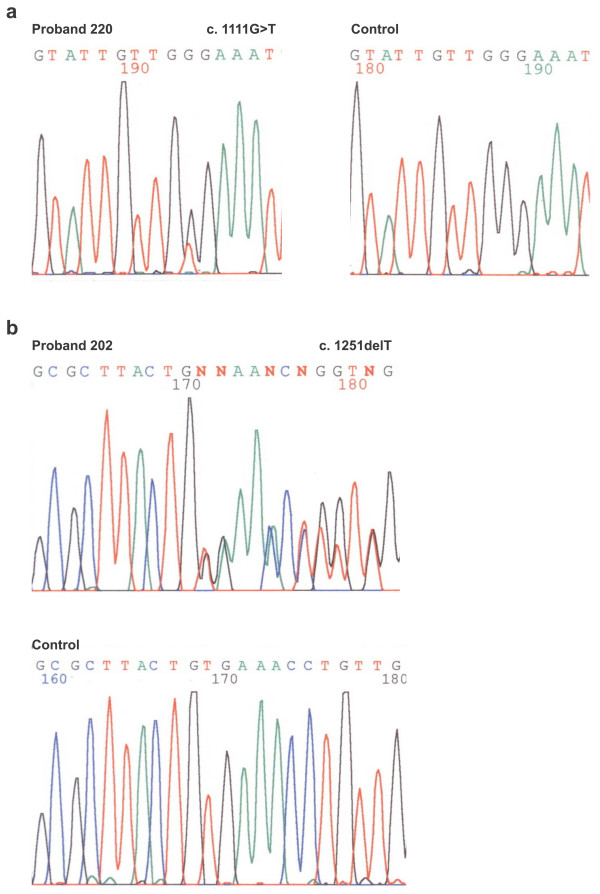
Analysis of *APC *germline mutations of exon 9. **a**. Sequencing pattern of alternatively spliced part of the exon 9 (forward) in proband of the family 220 (AFAP phenotype) with heterozygous substitution G>T at nucleotide position 1111 (p.Gly371X) and control **b**. Sequencing pattern of part in the regularly spliced region of the exon 9 (forward) in proband of the family 202 (FAP phenotype) with heterozygous deletion T at nucleotide position 1251 (p.Cys417TrpfsX37) and control.

The patients with germline *APC *mutations in the alternatively spliced exon 9 (probands 262, 220) have a phenotype of attenuated FAP (Fig. 2a). The proband 220 is a woman which developed < 100 polyps when she was 36 years old and then she is classified as AFAP. Her sister was diagnosed for polyp occurrence (< 100) at the age of 40 and her father was diagnosed for colorectal cancer at the age of 50. The patient 262 was found to have about 100 polyps at the age of 54. He had no cancer in the colon and his family history was negative. Variable phenotypes in the alternatively spliced region of exon 9 are described by Sieber et al. [22]. They analysed phenotypes and genetic pathways in attenuated FAP associated with germline mutations in the 5', 3'and exon 9 of the *APC *gene. Their genetic analysis revealed a requirement for "three hits" in at least some AFAP adenomas. In exon 9 mutant patients, the third hits were more frequent in adenomas than in 5'and/or 3'mutant patients.

The patients 202 and 215 with mutations localized in the regularly spliced region of exon 9 (Fig. 2b) are expected to present with classical FAP. Indeed, the patient 202 developed thousands of polyps at the age 30 and underwent total colectomy without evidence of cancer. The disease picture of the patient 215 is not so unambiguous. He was diagnosed for both polyps (hundreds) and cancer at the age 42. However, the later diagnosis can be connected with his negative family history or later occurrence of symptoms while polyp development could start several years earlier.

The possible explanation of variable FAP phenotypes may be other modifying genes, epigenetic mechanisms and environmental factors. Currently, reasons of attenuated course of the disease and a role of the AUG (start codon) at codon 184 [23] are searched and discussed [22].

The remainder patients with novel germline mutations in intron 5 (proband 164), exon 8 (proband 226) and 15 (probands 157, 233, 206 and 240) presented with the classical form of FAP with the > 100 polyps developed in the second and/or third decade of life (Tab. 1).

In a set of Slovak probands four novel mutations were discovered (Tab. 1). The newly described mutations were frameshift or nonsense in the 4 cases resulting in a truncated APC protein (probands S7, S16, S1 and S10). The mutation analysis revealed two DNA variations in the proband S10: the frame shift mutation (p.Asp1138ValfsX27) resulting in a truncating protein and one silent mutation which affected the first nucleotide of exon 15 (c.1959G>A, p.Arg653Arg) predicting the possibility of the aberrant splicing of the acceptor splice site in intron 14. However, this silent substitution rather represents a rare polymorphism because recently it was shown not to influence splicing on mRNA level [24]. All of the patients with the novel mutations presented the classical form of the disease in accordance with a localization of mutations except for the proband S7. This patient has the 29 bp deletion in exon 7 resulting in frameshift and premature stop codon. His phenotype varies between the FAP and AFAP with thousands of polyps without carcinoma but with later onset (at age 37) of the disease. This conclusion is supported by clinical pictures in his relatives. First symptoms occurred at 51 years of age in his father (diagnosis of CRC and thousands of polyps) and the similar manifestations occurred in his two brothers (CRC and thousands of polyps at 52 and 53 years of age respectively).

### Large *APC *deletions

Using MLPA analysis of 50 (24 probands from this paper and 26 patients from our previous studies) unrelated Czech and 3 Slovak FAP patients with no *APC *point mutation was performed. MLPA analysis revealed large deletions in 3 of the 50 Czech patients (6%). No large deletion was detected in Slovak patients. In Czech population, the frequency of large deletions appears to be slightly lower than recently published data [9,10]. Of the three deletions, the two whole-gene deletions including the promoter region (probands 43 and 54) and one exon 14 deletion were found. Microsatellite analysis was undertaken to determine the extent of the whole-gene deletions. On the basis of this analysis we can conclude that the distal breakpoint is situated between *APC *and the region of the D5S346, *MCC *gene and the deletion at the proximal site does not interfere in the region of the D5S299. The precise localization of the deletion breakpoints has not been finished. All three large deletions were identified in the set of patients with classical FAP, and not in patients suspected of AFAP confirming recently published data [9,10]. The FAP phenotype was quite classical with a hundred to thousand colorectal adenomas, CHRPE and with development of desmoid tumours and osteomas.

## Conclusion

In our study both novel and reported *APC *mutations were identified in Czech and Slovak FAP patients. The results of the molecular genetic testing are used both in the establishment of the predictive diagnosis and in the clinical management of patients. In some cases this study has also shown the difficulty to classify clinically between the classical and the attenuated form of FAP according to the established criteria: some clinical manifestations in a patient are characteristic for classical FAP and another feature resembles attenuated FAP in the same patient. Interfamilial and/or intrafamilial phenotype variability was also confirmed in some cases which did not fit well with predicted genotype-phenotype correlation. All these findings have to be taken into consideration both in the genetic counselling and in the patient care.

## Competing interests

The author(s) declare that they have no competing interest.

## Authors' contributions

JS carried out the molecular genetic studies, majority of mutation screening in *APC *and participated in the design of the study. MS, KZ carried out screening of large *APC *deletions. VK carried out sequencing of *APC *mutations. JK, KV, DI are clinical geneticists, they contacted the family members of the patients and collected clinical data and blood samples. MK conceived of the study, participated in its design and coordination and drafted the manuscript. All authors read and approved the final manuscript.

## Pre-publication history

The pre-publication history for this paper can be accessed here:


